# Unveiling the role of SYNGR4 in breast cancer development: a novel target for immunotherapy

**DOI:** 10.3389/fonc.2024.1490073

**Published:** 2025-01-20

**Authors:** Jie Ma, Hongtao Wang, Zhengwei Gui, Yuanrong Yang

**Affiliations:** ^1^ Department of Mammary, Jingzhou Hospital Affiliated to Yangtze University, Jingzhou, Hubei, China; ^2^ Department of Pharmacy, Jingzhou Hospital Affiliated to Yangtze University, Jingzhou, Hubei, China; ^3^ Department of Thyroid and Breast Surgery, Tongji Hospital of Tongji Medical College of Huazhong University of Science and Technology, Wuhan, Hubei, China

**Keywords:** breast cancer, SYNGR4, cell proliferation, immune infiltration, oncogene

## Abstract

**Introduction:**

SYNGR4 is considered to be one of the causative genes for amyotrophic lateral sclerosis, but its role in breast cancer development has not been revealed.

**Methods:**

The expression of SYNGR4 in a variety of malignancies including breast cancer was analyzed using Genotype Tissue Expression (GTEx) and the Cancer Genome Atlas (TCGA) databases and verified by specimens collected from our center. The effect of SYNGR4 on breast cancer prognosis was analyzed using bioinformatics and possible pathways by which this molecule affects breast cancer prognosis were explored. The effect of SYNGR4 on immune infiltration of breast cancer was analyzed using GSVA, and the effects of SYNGR4 on breast cancer proliferation, migration, and tumor-associated macrophage polarization in cancer foci were verified by cellular and animal experiments, respectively.

**Results:**

SYNGR4 is highly expressed in a variety of malignant tumors, including breast cancer, and affects the prognosis of breast cancer patients. This may be a volatile effect through Organelle fission, chromosome segregation, nuclear division, etc. SYNGR4 overexpression affects breast cancer proliferation, migration, and tumor immune infiltration, and promotes breast cancer tumor-associated macrophage polarization toward M2.

**Discussion:**

SYNGR4 overexpression can affect the prognosis of breast cancer patients by promoting M2 polarization of tumor-associated macrophages in breast cancer, and this molecule may be a novel target for breast cancer immunotherapy.

## Introduction

Breast cancer has become the most common cancer globally, surpassing lung cancer in recent years and posing a growing threat to the well-being of a larger population (1, 2). Conventional methods for treating breast cancer involve surgical procedures, radiation therapy, chemotherapy, hormone therapy, and targeted treatments (3–5). Early detection and consistent diagnosis and treatment have significantly enhanced the outlook for individuals with breast cancer (6, 7). However, the effectiveness of breast cancer therapy remains a major area for enhancement due to tumor diversity and resistance to medication (8, 9).

Immunotherapy is considered the most promising treatment for eliminating cancer, with numerous ongoing studies and clinical trials focused on breast cancer immunotherapy (10–12). Despite this, the limited effectiveness of current treatments has hindered the widespread adoption of breast cancer immunotherapy. The identification of novel immunotherapy targets is crucial in the age of precision medicine to enhance the outlook for individuals with breast cancer and reduce healthcare expenses (13).

SYNGR4 is a member of the SYNGR protein family, with SYNGR1 being predominantly found in neurons of the central nervous system and to a lesser extent in other tissues. It is believed to be linked to schizophrenia and bi-directional affective disorders. SYNGR2 is highly expressed in all tissues except the brain, and is believed to function as an oncogene in esophageal squamous cell carcinoma and hepatocellular carcinoma (14). SYNGR3 shows high levels of expression in the brain and is significantly upregulated in Parkinson’s disease and Alzheimer’s disease, with some evidence suggesting it may also be a contributing factor in Head and Neck Cancer (15).

SYNGR4 is believed to be the gene responsible for amyotrophic lateral sclerosis, but its involvement in breast cancer remains unclear.

## Materials and methods

### Clinical samples from patients

Jingzhou Central Hospital collected 10 paired cases of breast cancer tissues and paracarcinoma tissues from June 2023 to December 2023.The samples were stored in a negative 80degree Celsius environment. Written informed consent was obtained from all participants after approval by the Ethics Committee at Jingzhou Central Hospital.

### Survival analysis in the TCGA database

Based on the levels of SYNGR4 expression, breast cancer samples from the TCGA dataset were divided into groups with high and low expression. Kaplan-Meier plots were used to analyze the prognostic variances of these two groups. Functional enrichment analysis

We identified 1290 genes correlated with SYNG4 co-expression in BC using the clusterProfiler package in the R software (|FoldChange| > 1.5 and P <0.05). On the basis of the 1482 genes associated with the prognosis of BC, GO/KEGG pathway analysis was performed on the 104 genes, followed by visualization using Graphpad Prism.

### Cell culture and treatment

MCF7, MDA-MB-231 and SKBR3 cell lines were cultured in DMEM medium containing 10% fetal bovine serum, and MCF10A cell line was cultured in MCF10A special medium. All cell lines were cultured at 37°C in a humidified incubator with 5% CO2. The cell lines SKBR3, MCF-7, MDA-MB-231 and MCF-10A were purchased from Wuhan Institute of Cell Biology. All the purchased cell lines were identified by STR and compared with authoritative databases.

### Pathological sample processing

Tumors and surrounding tissues were exposed to a 10% formalin solution, then encased in paraffin, sliced into 5 mm sections, and underwent dewaxing, rehydration, and microwaving. Following a 30-minute incubation at 1°C, the samples were treated with SYNGR4 antibody from Thermofisher Scientific (PA5-20879) at room temperature. Subsequently, the secondary antibodies were labeled with DAB substrate and hematoxylin.

### RNA extraction and qRT-PCR

Total RNA was isolated using TRIzol reagent with SYNGR4 and GAPDH primers purchased from Tsingke Biotech. The primer sequences for SYNGR4 were as follows: forward ACCGACGGCTACCAGAACA and reverse GAGCTGGAAGGCTGTCTTGA.PCR was performed 40 times at 95°C for 5 minutes, then normalized to the internal control and analyzed using a 2-ΔΔCT approach.

### CCK8 assay

Digest the cells in logarithmic growth phase with trypsin digestion, with good growth status and collect and count them, calculate the appropriate amount of cell suspension according to the results of cell counting, and add complete medium, dilute 1.5~3.5×104 cells/mL, inoculate the cells in 96-well plates, add 100 uL of diluted cell suspension to each well, and inoculate six replicate wells in each group, and add in the wells around the inoculation area complete medium, repeat the inoculation of four 96-well plates, and incubated at 37°C in a carbon dioxide incubator, after inoculation, remove a 96-well plate every 24 hours, the remaining liquid medium was completely aspirated, 100 uL of serum-free medium containing 10% CCK8 was added to each well, and 100 uL of serum-free medium containing 10% CCK8 was added to the three empty wells as a blank control, and the well plate was The well plates were incubated in a carbon dioxide incubator at 37°C for 1 h, and the OD value at 450 nm was detected using an enzyme marker.

### Colony-formation assay

6-well plates were inoculated with a total of 5,000 cells. Following fixation in 4% polyacetal for 10 minutes, colonies were dyed with crystal violet. Photographs and tallies of colonies were taken.

### Transwell assay

Using a transwell chamber from Corning in the USA, 20,000 cells were removed from the upper surface after incubating for 24 hours at 37°C. Subsequently, the cells were stained with crystal violet for 10 minutes, followed by counting and imaging of the migrating cells.

### Scratch test

IBIDI dual-well culture inserts were placed in 24-well plates with SKBR3, MCF-7, and MDA-MB-231 breast cancer cells and incubated for 24 hours. The inserts were carefully extracted from the pristine surface using forceps, then 1mL of low serum media was added to each well. The wells were then observed under a light microscope on days 0 and 1 following insert removal.

### Tumor xenograft

A total of 12 female Balb/c mice, aged 8 weeks, with an average weight of 25 grams. A total of 1000 cells were implanted subcutaneously into the flanks of 6 mice per group. The xenografts were measured every other day to determine their final size. The final size was calculated as V = 0.5 × L (tumor length) × W^2^ (tumor width). We observed each nude mouse for 20 days, then anesthetized it with carbon dioxide until death, then excised the tumor and weighed it. This study was approved and conducted by the Animal Ethics Committee of Jingzhou Central Hospital. All operations were performed in accordance with the protocols stipulated by the Animal Ethics Committee of Jingzhou Central Hospital.

### Immune cell infiltration

Immune cell infiltration in BC was analyzed with the GSVA package in R, and the results were generated with the ssGSEA package. In TCGA-BRCA samples corresponding to high or low immunity cells infiltration, SYNGR4 expression was determined by categorizing 24 immune cells and consulting previous research.

### Immunofluorescence microscopy

DAPI staining and paraformaldehyde exposure for 15 minutes enabled the identification of actin and nuclei in cells. After staining the cells, they were examined under fluorescence microscopy.

### ELISA

In a coculture of BC cells and macrophages, levels of specific cytokines were measured by ELISA in the culture medium obtained from the lower chamber. We calculated the 450 nm absorbance using an enzyme marker and expressed it in picograms per milliliter using a standard curve.

## Results

### SYNGR4 expression analysis

**Figure 1:**
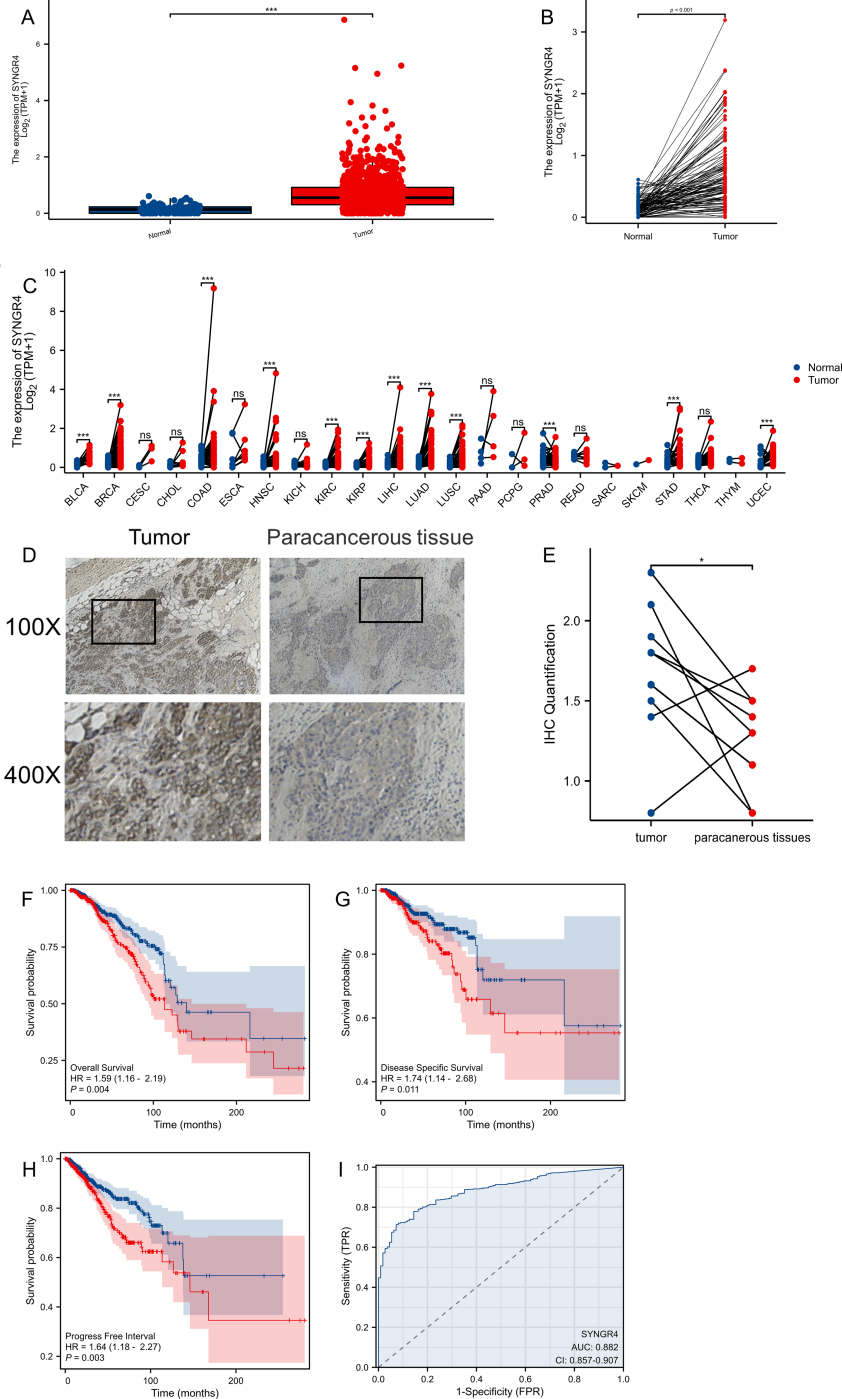
The expression difference and prognosis of SYNGR4 in breast cancer. **(A)** Expression of SYNGR4 in unpaired breast cancer samples in TCGA database. **(B)** Expression of SYNGR4 in paired breast cancer samples in TCGA database. **(C)** Expression of SYNGR4 in pan-cancer and adjacent normal tissues in TCGA and GTEx databases. **(D)** Typical cancer and paracancer SYNGR4 immunohistochemical images. **(E)** Quantitative SYNGR4 immunohistochemical analysis of 10 paired specimens. **(F)** OS of breast cancer patients based on SYNGR4 expression level. **(G)** DSS of breast cancer patients based on SYNGR4 expression level. **(H)** PFI of breast cancer patients based on SYNGR4 expression level. **(I)** Diagnostic ROC curve of SYNGR4. Data were shown as mean ± SD. ns: no significant, *p < 0.05, ***p < 0.001.

SYNGR4 was found to be upregulated in breast cancer samples, both unpaired and paired, based on data from the TCGA and GTEx pancancer databases (**Figures 1A, B**). In the pan-cancer analysis, SYNGR4 was overexpressed in 12 malignant tumors including breast cancer. (**Figure 1C**) This result was verified by the paired samples of breast cancer and paracancer collected at our center. Typical immunohistochemical pictures and quantitative analysis are shown in **Figures 1D, E**.

### Breast cancer patients’ prognosis and SYNGR4 expression

**Figure 2:**
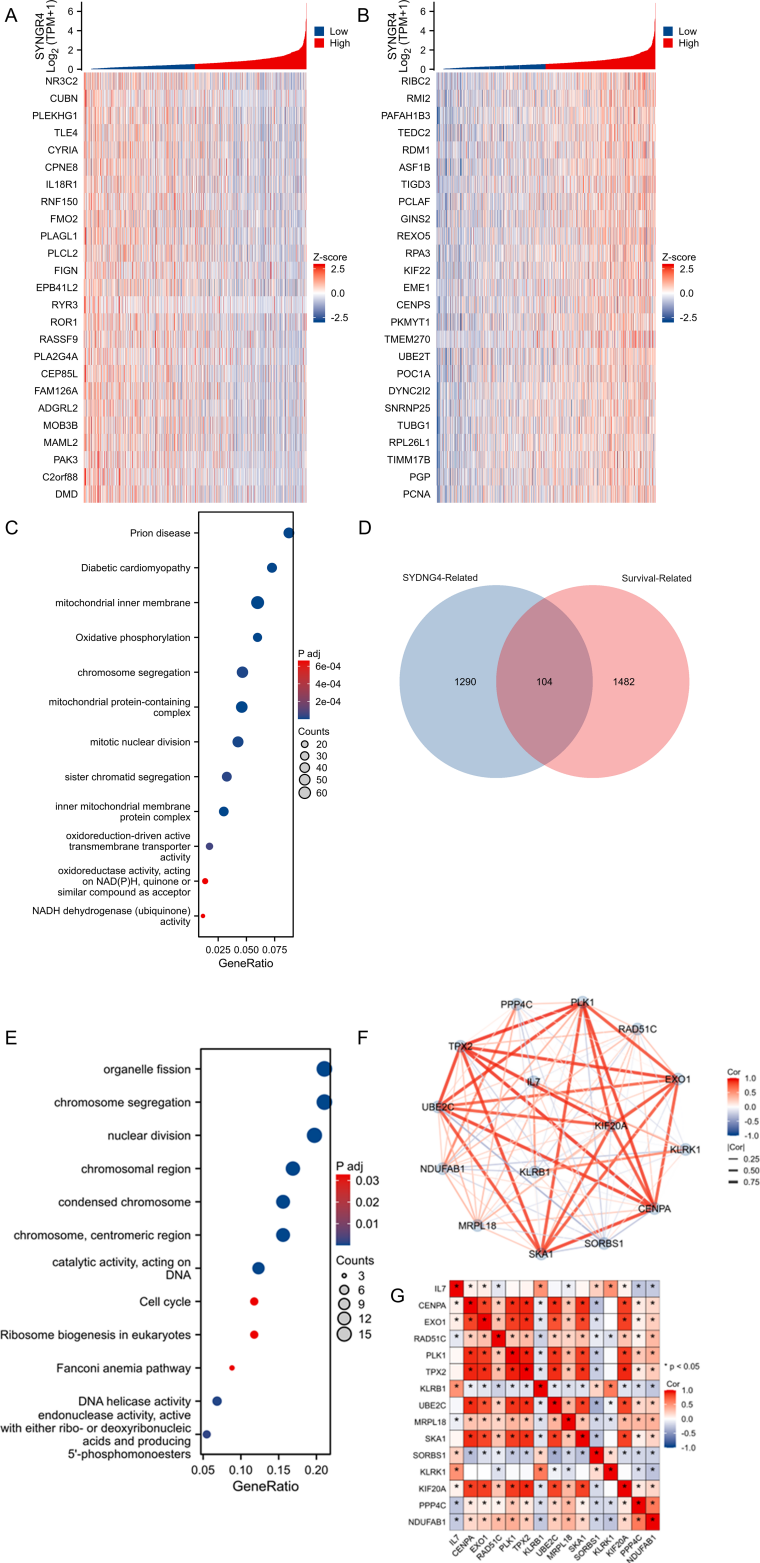
Analysis of SYNGR4-related enrichment pathways and molecular network analysis. **(A)** Heatmap of TOP25 genes positively associated with SYNGR4 co-expression. **(B)** Heatmap of TOP25 genes negatively associated with SYNGR4 co-expression. **(C)** GO/KEGG analysis of SYNGR4 co-expressed genes. **(D)** Wayne plot of SYNGR4 co-expressed genes taking intersection with BC survival related genes. **(D)** GO/KEGG analysis of SYNGR4 co-expressed genes. **(E)** GO/KEGG analysis of intersecting genes. **(F)** STING protein interaction network analysis. **(G)** Highest confidence molecular correlation heatmap.

We examined how SYNGR4 impacts the outlook for breast cancer by studying its correlation with OS (overall survival) (HR=1.59, P=0.004) (**Figure 1F**), DSS (disease specific survival) (HR=1.74, P=0.011) (**Figure 1G**), and PFI (progress free interval) (HR=1.64, P=0.003) (**Figure 1H**) in patients with breast cancer. Correlations were observed between OS (overall survival) (HR=1.64, P=0.003) and PFI (progression-free interval) (**Figure 1I**). **Figure 2D** shows the ROC curve for SYNGR4-related breast cancer diagnosis. (AUC=0.882).

### Correlation and enrichment analyses

SYNGR4 co-expressed genes were identified in the TCGA-BRCA dataset, and the top 25 genes showing positive and negative correlations were visualized in heat maps labeled as **Figures 2A**, **B**, correspondingly. Gene sets with absolute values greater than 1.5 and P values less than 0.05 were chosen as co-expressed genes for GO/KEGG analysis, with the findings presented in **Figure 2C**. Then, the genes in the TCGA-BRCA database were selected, duplicate samples were removed, and samples without clinical information were removed. The median gene expression was used as the cut-off value to divide the patients into two groups, and the Hazard Ratio (HR) was calculated based on the prognostic data of the high-expression and low-expression groups. The HR was arranged in descending order, and an absolute value greater than 1.5 and P less than 0.05 was taken to define the breast cancer prognosis-related gene group. Co-expressed genes and molecular genes associated with breast cancer prognosis-related molecular gene sets were taken to intersect (**Figure 2D**), 104 genes were obtained, and then GO/KEGG analysis was performed (**Figure 2E**), and the results suggested that the possible pathways by which SYNGR4 affects the prognosis of breast cancer patients are Organelle fission, chromosome segregation, nuclear division, etc. These 104 genes were selected for protein interactions analysis, and 15 molecules were screened by selecting the highest confidence level to visualize their protein interactions network (**Figure 2F**) and correlation heatmap (**Figure 2G**). This network may be a collection of molecular pathways of SYNGR4 affecting breast cancer prognosis.

**Figure 3:**
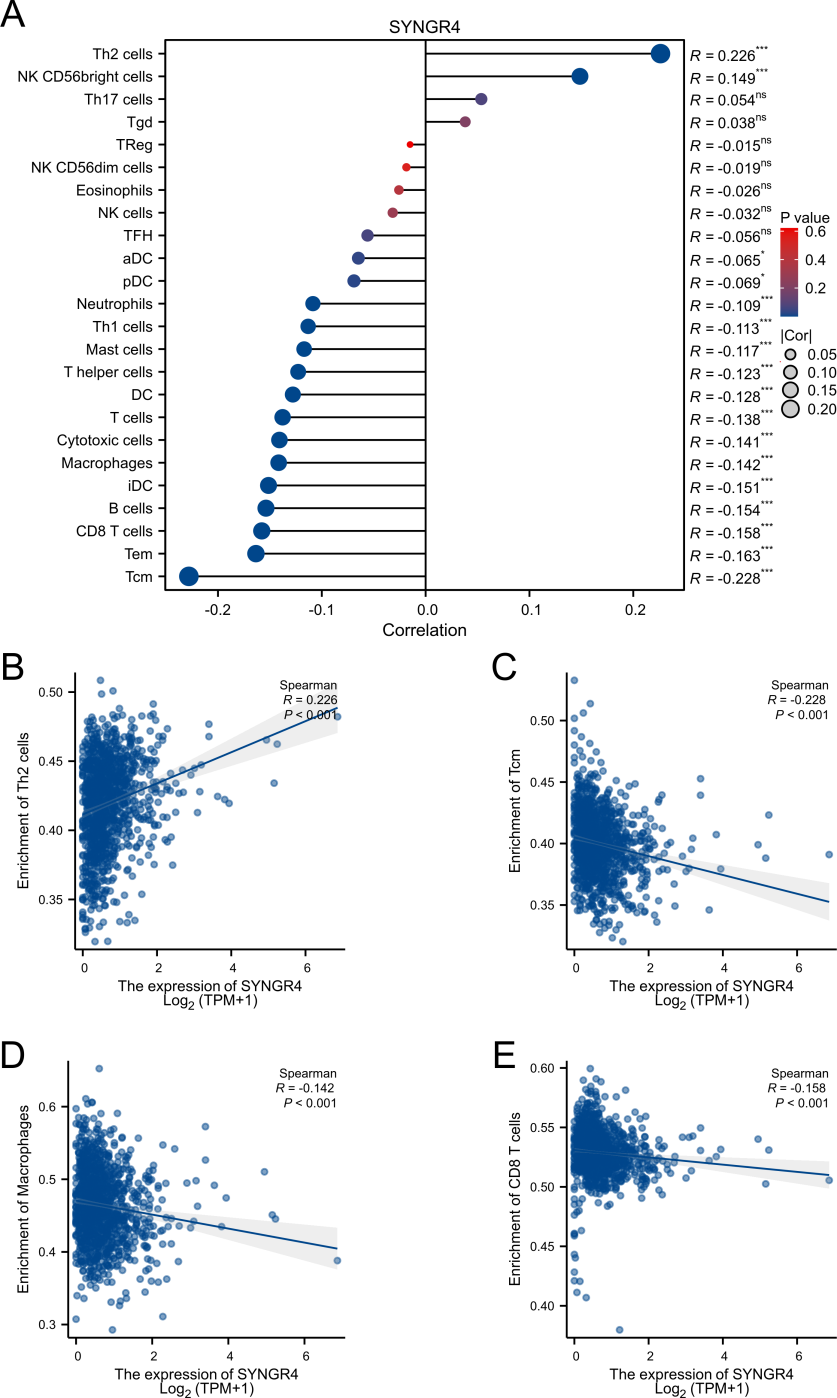
Associated between SYNGR4 with immune cell infiltration. **(A)** Correlation between the expression level of SYNGR4 and various immune cell infiltration. **(B)** Correlation between SYNGR4 expression and Th2. **(C)** Correlation between SYNGR4 expression and Tcm. **(D)** Correlation between SYNGR4 expression and Macrophages. **(E)** correlation between SYNGR4 expression and CD8+T cells, ns: no significant. *p < 0.05, ***p < 0.001.

### Expression of SYNGR4 and immune cell infiltration

**Figure 4:**
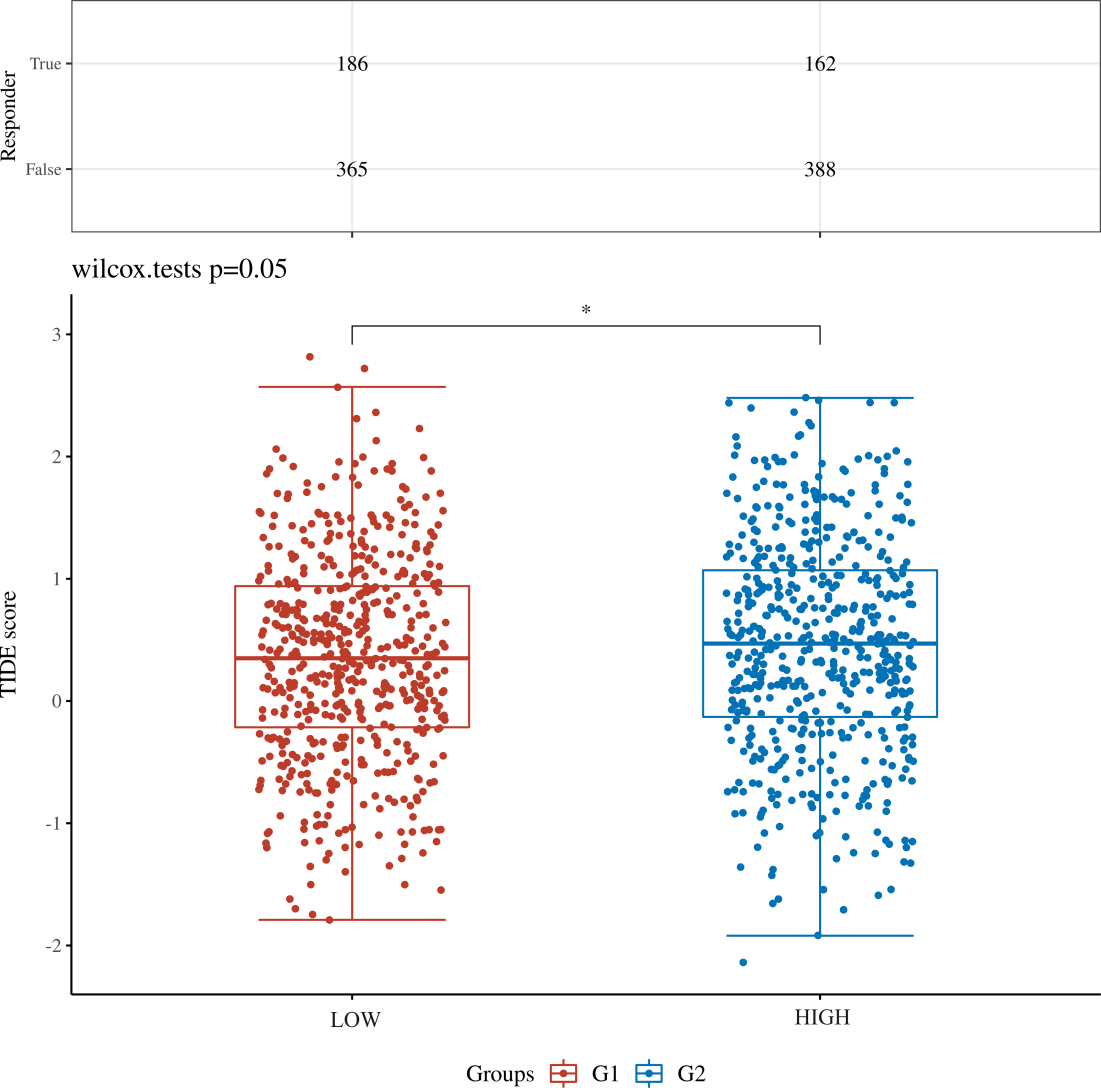
TIDE algorithm predicts the relationship between SYNGR4 expression and breast cancer immunotherapy responsiveness. *p < 0.05.

Patients from the TCGA-BRCA dataset were split into two categories based on median SYNGR4 levels for studying immune cell infiltration. **Figure 3A** displays the infiltration of 24 immune cells. With SYNGR4 overexpression, Th2 cell infiltration increased (**Figure 3B**), while Tcm (**Figure 3C**), macrophages (**Figure 3D**), and CD8-positive T cell infiltration decreased. (**Figure 3E**) Based on the TIDE algorithm to predict the responsiveness of breast cancers with different SYNGR4 expression groups to immunotherapy, breast cancers with SYNGR4 overexpression had a higher responsiveness to immunotherapy (**Figure 4**).

### Knocking down SYNGR4 inhibited malignant behavior in breast cancer cells

**Figure 5:**
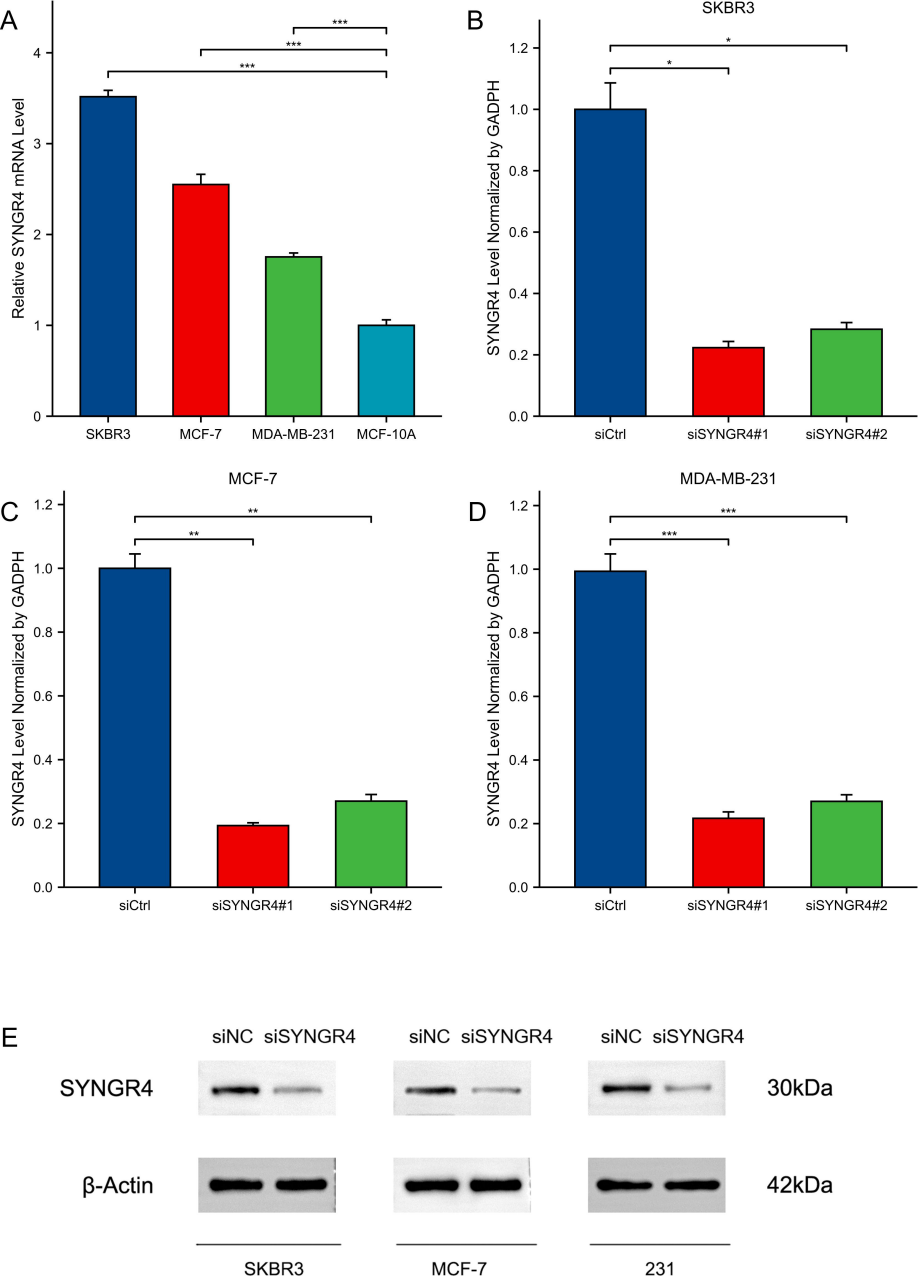
Expression and knockdown of SYNGR4 in various cell lines. **(A)** SYNGR4 expression in SKBR3, MCF-7, MDA-MB-231 and MCF-10A cell lines. **(B)** SYNGR4 knockdown efficiency of two siRNA in SKBR3 cell lines. **(C)** SYNGR4 knockdown of two siRNA in MCF-7 cell lines Efficiency. **(D)** SYNGR4 knockdown of two siRNA in MDA-MB-231 cell lines Efficiency. **(E)** Western blot shows successful knockdown of SYNGR4 at the protein level. *p < 0.05, **p < 0.01, ***p < 0.001.

**Figure 6:**
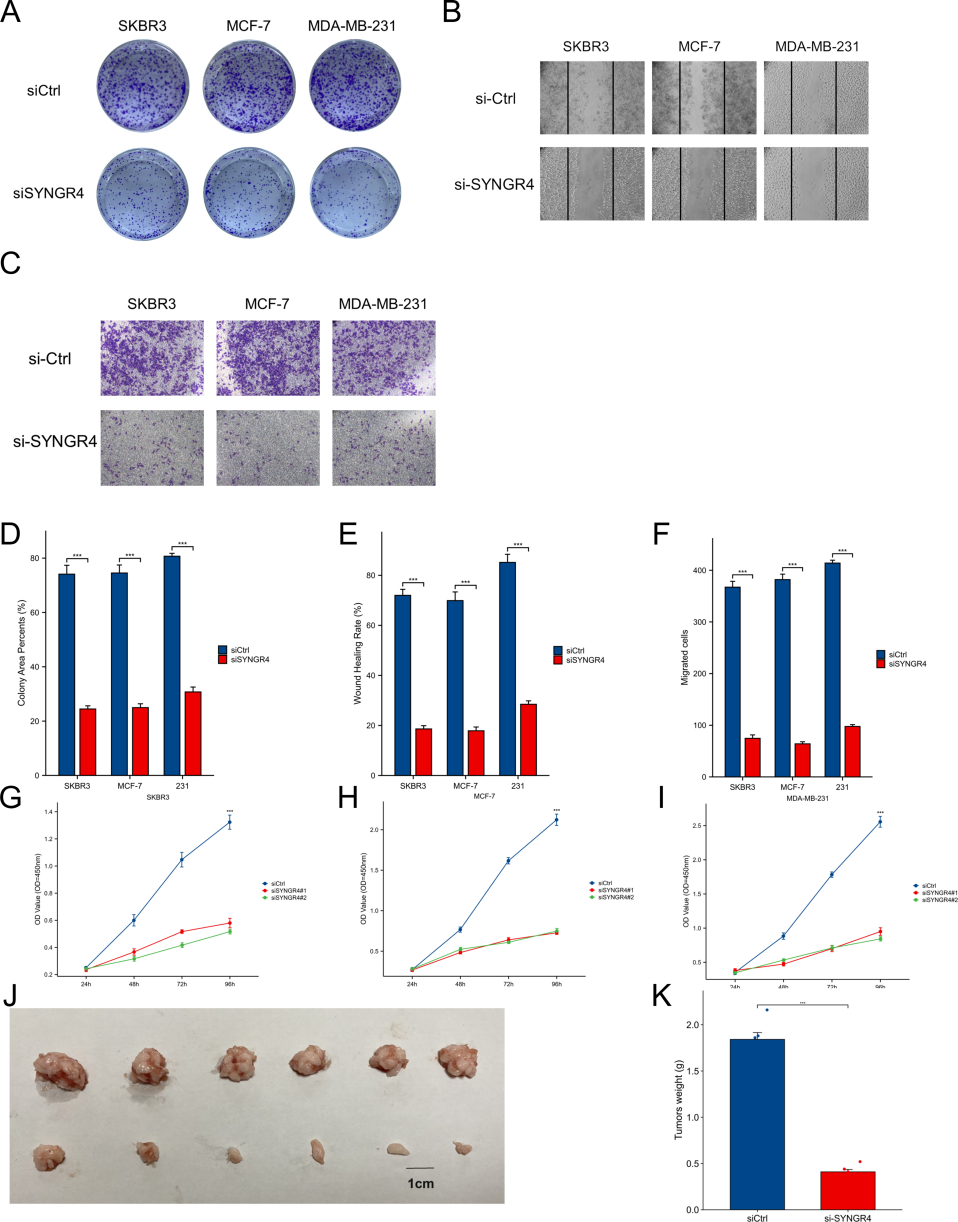
Cellular and animal experiments validate the effect of SYNGR4 on breast cancer cells. **(A)** Clone formation of control group and knockout groups in BC lines. **(B)** Scratch test images of control group and knockout groups in BC lines. **(C)** Transwell images of control group and knockout groups in BC lines. **(D)** Quantitative analysis of clone formation experiment. **(E)** Quantitative analysis of scratch experiment. All assays were independently repeated at least three times. **(F)** Quantitative analysis of transwell experiment. **(G–I)** Cell proliferation in knockout groups and control groups in BC cell lines. **(J)** Growth of transplanted tumors in nude mice injected with 231 cells and si-SYNGR4 231 cells. **(K)** Growth curve of grafted tumor volume. Data are presented as the mean ± SD. ***p < 0.001.

SYNGR4 showed significantly higher levels of expression in SKBRE3, MCF-7, and MDA-MB-231 cell lines compared to the MCF-10A cell line, which serves as a model for normal breast cells (**Figure 5A**). Knockdown of SYNGR4 in three breast cancer cell lines using two si-RNAs yielded satisfactory knockdown efficiencies. SYNGR4 knockdown decreased the malignant characteristics of breast cancer cells, including proliferation and migration, as shown in the clone formation assay (**Figures 5B–D**). In addition, the Western blot results likewise validated the successful knockdown of SYNGR4 at the protein level (**Figure 5E**). Colony-formation assay (**Figure 6A**), Transwell assay (**Figure 6B**), and scratch assay (**Figure 6C**). Multiple repetitions of the aforementioned experiments were conducted and subsequently subjected to statistical analysis (**Figures 6D–F**). CCK8 assay shows that knockdown of SYNGR4 significantly inhibits the proliferative capacity of various breast cancer cell lines (**Figures 6G–I**). *In vivo* experiments showed that SYNGR4 knockdown breast cancer had reduced tumorigenicity *in vivo* (**Figures 6J, K**).

SYNGR4 knockdown promotes tumor-associated macrophage M1 polarization in breast cancer

**Figure 7:**
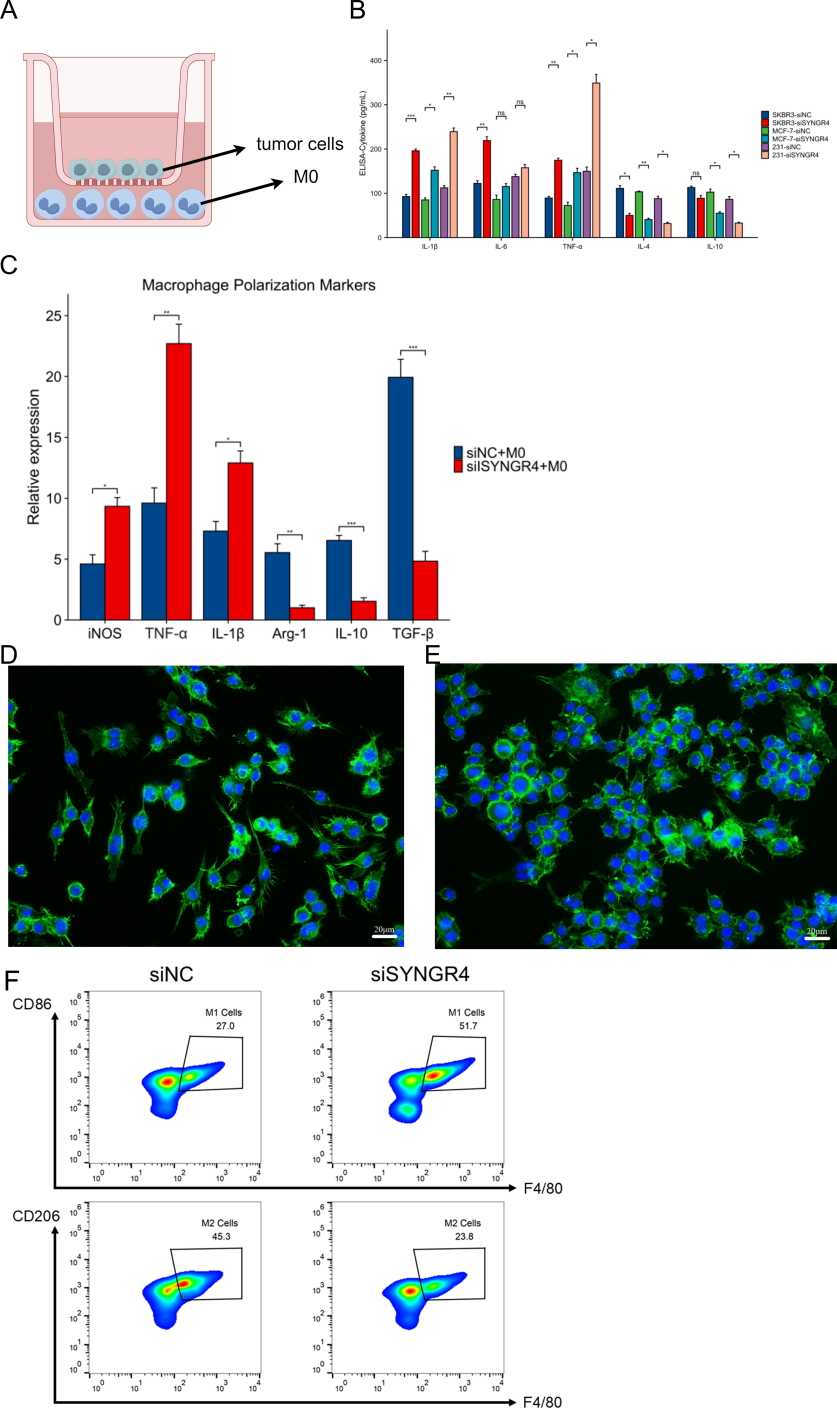
Effect of SYNGR4 knockdown on breast cancer tumor-associated macrophages. **(A)** Schematic diagram of the co-culture model of breast cancer cells and macrophages. **(B)** Cytokine content (ELISA) of culture fluid in co-culture chambers. **(C)** Macrophage polarization marker expression. **(D)** Immunofluorescence graph of macrophage morphology (si-Ctrl). **(E)** Immunofluorescence graph of macrophage morphology (si-SYNGR4). **(F)** Macrophage polarisation in control and SYNGR4 knockdown groups detected by flow cytometry.

To mimic the environment of tumor immune microenvironment in which breast cancer cells interact with tumor-associated macrophages, we constructed a co-culture model as shown in **Figure 7A**. After 36 hours of co-culture, the lower chamber’s cell culture fluid was gathered for cytokine level assessment. Compared to the control group, pro-inflammatory cytokines like IL-1β, IL-6, and TNF-α increased, while anti-inflammatory cytokines IL-4 and IL-10 decreased in the SYNGR4 knockdown group (**Figure 7B**). Macrophages were collected and assayed for the expression of polarization markers in them. iNOS, a marker of M1 activation, along with TNF-α and IL-1β, showed increased levels in the group with SYNGR4 knockdown, whereas Arg-1, IL-10, and TGF-β, markers of M2 activation, exhibited decreased levels. Immunofluorescence maps showed that macrophages in the SYNGR4 knockdown group were in the M1-polarized morphology, while the control group tended to be in the M2-polarized morphology (**Figures 7C–E**). To further validate the effect of SYNGR4 on macrophage polarization in the tumor immune microenvironment, we analyzed macrophages in the tumors of mice in the SYNGR4 knockdown and control groups by flow cytometry, and the results showed an increased polarization of macrophages toward M1 and a significant decrease in M2-like macrophages in breast cancers of mice with SYNGR4 knockdown relative to controls (**Figure 7F**).

## Discussion

The frequency of breast cancer has significantly increased in the last forty years. In 2040, over 3 million new instances of breast cancer are anticipated annually, resulting in more than 1 million fatalities each year (16). After decades of continuous research, researchers have developed a variety of new therapies in addition to traditional treatments for breast cancer. PARP inhibitors like Olaparib (17), CDK4/6 inhibitors such as palbociclib (18), abemaciclib (19), and ribociclib (20) have received FDA approval for treating breast cancer. Clinical trials are currently testing AKT inhibitors like MK-2206 (21) and angiogenesis inhibitors like bevacizumab. However, the optimal treatment for all subtypes of breast cancer has not yet been identified. Hence, identifying novel genes responsible for breast cancer and finding new targets for treatment are crucial for effectively treating breast cancer.

With advances in immunology, there is a growing understanding of tumor escape immune cell surveillance and killing. Recent recognition of immunotherapy as a potential treatment for breast cancer involves targeting specific proteins expressed in cancer cells, showing promise for therapy. Immune checkpoints can influence T cell activation and tolerance (22, 23). Inhibitory signals from the PD-1/PD-L1 axis and CTLA-4 can hinder T-cell immune responses, preventing the elimination of tumor cells and potentially promoting tumor growth (24). Drugs like Pembrolizumab, Nivolumab, Avelumab, Atezumab, and Durvalumab have been created for breast cancer immunotherapy, but their effectiveness is restricted by PD-1/PD-L1 expression, limiting the number of patients who can benefit from them (25, 26).

Increasing understanding of the tumor immune microenvironment has led to the realization that the interaction between tumors and immune cells within cancer foci may offer a new approach for treating cancer. Our study revealed that elevated levels of SYNGR4 led to enhanced infiltration of Th2 cells in the immune microenvironment of breast cancer. Both Th1 and Th2 cells originated from the precursor cell Th0, with increased Th2 cells capable of producing various cytokines like IL-4 and IL-10. This created an anti-inflammatory environment that facilitated tumor evasion from immune destruction. Meanwhile, the reduction of Tcm (Central Memory T cell) and macrophages, CD8-positive T cells can reduce the long-term and short- and medium-term tumor killing ability of immune cells, respectively. Thus, SYNGR4 has the potential to reverse this Th2/Th1 drift and T cell depletion. In this study, we found that reducing SYNGR4 expression leads to a shift in tumor-associated macrophages towards the M1 (pro-inflammatory) phenotype in breast cancer, potentially aiding in the reversal of the anti-inflammatory tumor immune microenvironment. Macrophages, originating from monocytes, are crucial for both innate and adaptive immunity. It should be noted that a co-culture model of tumor cells and macrophages was used in our study to simulate the interaction between tumor cells and immune cells in the tumor immune microenvironment; however, there are some differences between this simple co-culture model and the real immune microenvironment. New *in vitro* 3D models (27) and derived models such as organoids (28, 29) may be able to better mimic the real *in vivo* environment, which will be the effort of our next work.

Overall, SYNGR4 could be a promising target for breast cancer immunotherapy and deserves further investigation.

The shortcoming of this study is that, although a preclinical model of co-culture was developed to validate the mechanism of action of SYNGR4, there are discrepancies between the co-culture model and the real, complex tumor immune microenvironment, which, on the other hand, differs to some extent from that in humans in mice, and thus further *in vivo* experiments are needed to assess the therapeutic potential of SYNGR4.

## Data Availability

The original contributions presented in the study are included in the article/supplementary material. Further inquiries can be directed to the corresponding author.
